# Associations Between Makeup Use and Physical, Cognitive, and Psychological Functions in Community-Dwelling Older Women

**DOI:** 10.3390/healthcare13202618

**Published:** 2025-10-17

**Authors:** Shinya Matori, Shin Murata, Yuki Kikuchi, Hideki Nakano, Takeshi Katsurasako, Kohei Iwamoto, Kohei Mori, Akio Goda, Kenji Kamijo

**Affiliations:** 1 Graduate School of Health Sciences, Kyoto Tachibana University, Kyoto 607-8175, Kyoto, Japan; katsurasako@tachibana-u.ac.jp (T.K.); h901523001@st.tachibana-u.ac.jp (K.I.); 2 Department of Occupational Therapy, Nursing & Rehabilitation Ryokuseikan, Tosu 841-0074, Saga, Japan; 3 Department of Physical Therapy, Faculty of Health Sciences, Kyoto Tachibana University, Kyoto 607-8175, Kyoto, Japan; murata-s@tachibana-u.ac.jp (S.M.); kickpt4018@outlook.com (Y.K.); nakano-h@tachibana-u.ac.jp (H.N.); 4 Faculty of Allied Health Sciences, Kansai University of Welfare Sciences, Kashiwara 582-0026, Osaka, Japan; kmori@tamateyama.ac.jp; 5 Department of Physical Therapy, Faculty of Health and Medical Science, Hokuriku University, Kanazawa 920-1180, Ishikawa, Japan; a-goda@hokuriku-u.ac.jp; 6 Department of Rehabilitation, Faculty of Wakayama Health and Medical Sciences, Takarazuka University of Medical and Health Care, Wakayama 640-8392, Wakayama, Japan; k-kamijo@tumh.ac.jp

**Keywords:** older women, makeup, dynamic balance, depressive symptoms, quality of life

## Abstract

**Background/Objectives:** Older women who habitually wear makeup exhibit better cognitive and psychological functioning. However, physical characteristics associated with habitual makeup use in this population remain unclear. We aimed to evaluate physical, cognitive, and psychological characteristics of community-dwelling older women who habitually use makeup. **Methods**: This health survey included 295 community-dwelling women aged ≥65 years. Weekly makeup use frequency; cosmetic types used; grip strength; sit-and-reach distance; one-leg standing time; maximum walking speed; and scores on timed up-and-go (TUG) test, Mini-Mental State Examination, Geriatric Depression Scale-5 (GDS-5), and EuroQol 5-Dimension 5-Level (EQ-5D-5L, Japanese version) were statistically analyzed and compared between makeup and non-makeup groups. Further, the following four groups, based on lipstick and eyebrow product use, were compared: lipstick users, eyebrow products users, both-users, and neither-users. **Results**: The make-up group had lower age (*p* = 0.001), lower TUG scores (*p* = 0.011), lower fastest walking speed (*p* = 0.022), and lower GDS-5 scores (*p* = 0.009) and higher grip strength (*p* = 0.011), one leg standing time (*p* = 0.008), and EQ-5D-5L scores (*p* = 0.049). After adjusting for age, the make-up group showed significantly lower GDS-5 scores (*p* = 0.008) and higher EQ-5D-5L scores (*p* = 0.038). Comparison by cosmetic types revealed significantly lower age (*p* = 0.004) and TUG (*p* = 0.007), GDS-5 (*p* = 0.002), and EQ-5D-5L (*p* = 0.034) scores and higher EQ-5D-5L scores in users than in non-users. Multinomial logistic regression analysis revealed a significant association with TUG (odds ratio [OR], 0.653; 95% confidence interval [CI], 0.448–0.952) and GDS-5 (OR, 0.592; 95% CI, 0.415–0.843) in both-users. **Conclusions**: Habitual lipstick and eyebrow cosmetic use may be associated with improved mood, quality of life, and dynamic balance in older women.

## 1. Introduction

The proportion of older adults in Japan has been steadily increasing owing to the declining birth rate and aging population. Consequently, the extension of healthy life expectancy and implementation of preventive care strategies have become major issues in Japan [1]. Individualized strategies aimed at restoring the physical function of older people are beneficial; however, the promotion of social participation and enhancement of quality of life (QOL) are essential components of long-term preventive care [2]. Older adults are particularly vulnerable to social isolation resulting from retirement, spousal bereavement, or the loss of social roles [3,4], all of which increase the risk of depression [5] and withdrawal from society [6]. Therefore, the development of strategies that encourage physical activity and social engagement among older adults is important for improving and maintaining their QOL.

Makeup or cosmetic use is a meaningful activity that may promote social participation among older women. Cosmetics are defined as “articles intended to be rubbed, poured, sprinkled, or sprayed on; introduced into; or otherwise applied to the human body for cleansing, beautifying, and promoting attractiveness or altering physical appearance” [7]. They are broadly categorized into skincare products—including lotion, milky toners, and essences—and makeup products, such as foundation, lipstick, and eyebrow cosmetics [8]. The application of makeup stimulates the senses of touch, smell, and sight, thereby eliciting psychological pleasure [9]. Despite its widespread use, makeup tends to be worn less frequently as women age [10].

Previous studies have indicated that makeup application requires a certain level of hand dexterity and that older women who habitually wear makeup demonstrate better frontal lobe function than those who do not [11]. Moreover, habitual makeup use has been associated with a lower prevalence of depression [12] and greater willingness to engage in outdoor activities [13]. These findings suggest that habitual makeup use among older women contributes not only to the maintenance and enhancement of cognitive and psychological functions but also to increased social participation [14] and physical activity.

Most previous studies on makeup use among older women have primarily focused on its relationship with cognitive and psychological function. By contrast, the physical characteristics of older women who habitually use makeup have not been comprehensively examined. A clearer understanding of the physical functions associated with habitual makeup use may aid in the development of interventions to promote and sustain social participation and strategies to prevent care dependency in this population. Therefore, this study aimed to evaluate habitual makeup use among community-dwelling older women and examine the physical, cognitive, and psychological characteristics associated with this practice.

## 2. Materials and Methods

### 2.1. Participants

**Figure 1:**
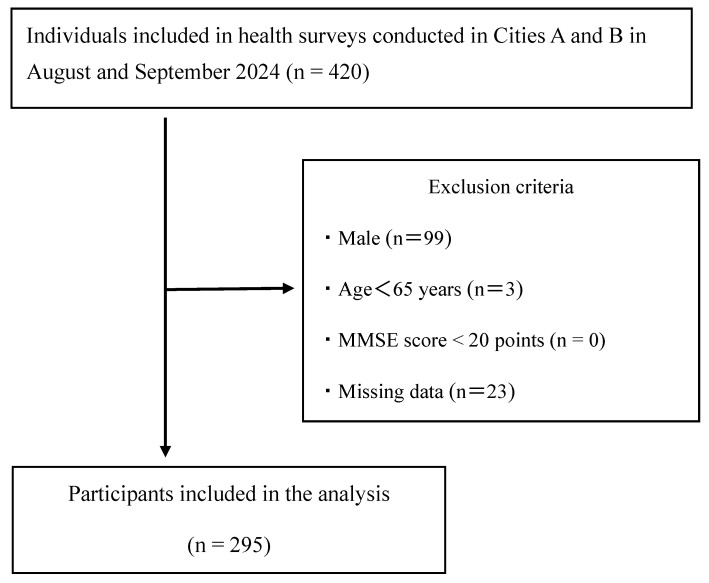
Flowchart illustrating the participant selection process. MMSE, Mini-Mental State Examination.

A total of 420 individuals were recruited to participate in health surveys conducted in Cities A and B in August and September 2024. Recruitment was conducted using posters and newsletters created by each region’s Comprehensive Community Support Center, and interested participants applied independently. The participants were elderly individuals who were able to reach the venue independently on foot or by bicycle, car, or public transportation and who were self-sufficient in their daily lives. The exclusion criteria were as follows: (1) male (*n* = 99), (2) age < 65 years (*n* = 3), (3) Mini-Mental State Examination (MMSE) score < 20 (*n* = 0) [15], and (4) missing data (*n* = 23). After excluding ineligible individuals, 295 participants were included in the final analysis (Figure 1).

### 2.2. Measurements and Procedures

#### 2.2.1. Current Makeup Usage Status

Skincare and makeup usage over the past week were assessed using a self-administered questionnaire. Skincare usage was determined by asking the question, “Do you use skincare (e.g., lotion, milky toner, or essence)?” The response options were as follows: “almost every day (≥4 days/week),” “sometimes (2–3 times/week),” and “rarely.” Participants who responded “almost every day” or “sometimes” were categorized as “skincare users,” whereas those who responded “rarely” were categorized as “non-skincare users.”

Makeup usage status was assessed by asking the question, “Do you wear makeup?” with the same response options as those for skincare usage. Participants who selected “almost every day” or “sometimes” were classified as “makeup users,” whereas those who selected “rarely” were categorized as “non-makeup users.” In addition, participants were asked to indicate the types of cosmetics they used daily by selecting from the following options: foundation, lipstick, eyebrow products, eye shadow, and blush. Previous studies have reported that older women use “lipstick” and “eyebrow products” to enhance their attractiveness and youthfulness [16,17]. This suggests that older adults who use “lipstick” and “eyebrow products” are more socially engaged and maintain better health. Therefore, among the aforementioned cosmetics, this study focused on the characteristics of older adults who use “lipstick” and “eyebrow products.” Participants who selected either lipstick or eyebrow products were classified into the corresponding groups. Participants who selected both products were categorized as “both-users,” whereas those who selected neither were classified as “neither-users.” We also determined whether the makeup was applied independently by the individual or with assistance by searching for relevant questions and responses in the survey.

#### 2.2.2. Physical Function

Physical function was evaluated by assessing grip strength, static balance, dynamic balance, flexibility, and gait ability. Grip strength [18] was measured using a digital dynamometer (T.K.K. 5401, Takei Kiki Kogyo Co., Ltd., Niigata, Japan). The participants stood with their arms at their sides, index fingers pointing forward, and the grip adjusted so that the second finger formed a 90° angle. Two measurements were taken for each hand, and the maximum value from each side was recorded.

Static standing balance was assessed by measuring one-leg standing time with the eyes open [19]. The participants were instructed to raise the non-supporting foot forward while placing both hands on the hips. Timing began once the correct posture was assumed. The test was terminated if the raised foot touched the supporting leg or the floor, the supporting foot shifted, or either hand was removed from the hips. Each attempt was limited to a maximum of 120 s and was performed twice on each leg. The longest time recorded was used for the analysis.

Dynamic balance was assessed using the timed up-and-go (TUG) test, following the method described by Wada et al. [20]. The test began with the participant seated in a 40-cm-high chair without armrests, with both upper limbs resting on the thighs and both feet placed side by side with toes aligned. Upon the cue, the participant stood up, walked around a cone placed 3 m in front of the chair, and returned to seated position. The time required to complete the sequence was measured using a stopwatch. The participants were instructed to perform the task with maximum effort. The test was performed twice, and the shortest time was used for the analysis.

Flexibility was assessed using the sit-and-reach test. The distance reached was measured using a digital sit-and-reach measurement device (T.K.K. 5412, Takei Kiki Kogyo Co., Ltd.). The participant began in a seated position with the back of the head and lower back firmly against the wall, both feet together, and the knees fully extended. They were instructed to place both hands on the device with elbows extended and to lean forward as far as possible [19]. The measurements were taken twice, and the longest distance was recorded as the representative value.

Gait ability was assessed by measuring maximum walking speed using a sheet-type device that records foot pressure, ground contact, and footprints (Walk Way MW-1000, Anima Co., Ltd., Tokyo, Japan) [21]. The measurement sheet was 2400 mm in length and 800 mm wide, with a spatial resolution of 10 × 10 mm and 14,400 measurement points. The walking path included a 2 m approach zone before and after the 2.4 m measurement section. The participants were barefoot and instructed to “walk as fast as possible without running.” The trials were conducted, and the fastest speed was used for the analysis.

#### 2.2.3. Cognitive Function

Cognitive function was assessed using the MMSE [22]. This tool is widely used for measuring cognitive function. It consists of 11 items, including orientation to time and place, immediate and delayed recall of three words, calculation, object naming, sentence repetition, execution of three-step verbal commands, following written instructions, sentence writing, and figure copying. The maximum score is 30 points, with a score below 20 points indicative of cognitive impairment suggestive of dementia [15].

#### 2.2.4. Psychological Function

Psychological function was assessed by evaluating depressive symptoms using the Geriatric Depression Scale-5 (GDS-5) [23]. This questionnaire asks respondents to provide “yes” or “no” responses to five items related to depression, and its validity and reliability have been confirmed [24]. One point is awarded for each affirmative response, and the total score across all items is used to assess the overall severity of symptoms [25].

Health-related quality of life (HRQOL) was assessed using the Japanese version of the EuroQol-5 Dimension-5 Level (EQ-5D-5L) questionnaire [26]. This instrument is used for evaluating HRQOL across five dimensions: mobility, personal care, usual activities, pain/discomfort, and anxiety/depression. Each item is rated on a five-point scale (none, some, moderate, severe, and extreme), with higher scores indicating better perceived health status. Responses were converted to a single index value using a Japanese scoring algorithm, which ranges from −0.025 to 1.000 [27].

### 2.3. Statistical Analysis

The participants were categorized into two groups based on their use of basic skincare products: skincare and non-skincare users. They were also classified as makeup users and non-makeup users according to their makeup usage habits. The number of participants in each group was tabulated. Comparisons of physical, cognitive, and psychological outcomes—including grip strength; one-leg standing time; TUG; sit-and-reach distance; maximum walking speed; and MMSE, GDS-5, and EQ-5D-5L scores—were carried out between the two groups using independent *t*-tests and analysis of covariance (ANCOVA), with age included as a covariate.

Makeup users were further subdivided into four groups based on the type of cosmetics used: lipstick only, eyebrow product only, both lipstick and eyebrow products, and neither. One-way analysis of variance (ANOVA) was performed to compare the outcome measures across these four groups. Significant differences were analyzed using post hoc tests, which were conducted using Tukey’s method. We also performed a multinomial logistic regression analysis to identify factors related to the type of cosmetics used as the dependent variable. Independent variables included age, maximum hand grip strength, MMSE score, and items showing significant differences in the one-way ANOVA. To account for multicollinearity, we calculated the variance inflation factor (VIF) and confirmed that the variance was less than five. Descriptive statistics are presented as mean ± standard deviation. Because the primary analysis was conducted using analysis of covariance (ANCOVA) with age as a covariate, parametric statistical methods were adopted for consistency. All statistical analyses were performed using SPSS version 29.0 (IBM Corp., Armonk, NY, USA). *p*-value of <0.05 was considered significant.

### 2.4. Ethical Considerations

This study was approved by the Research Ethics Committee of Kyoto Tachibana University (22-23 61). All participants received written and oral explanations of the study objectives and procedures prior to providing informed consent. This study was conducted in accordance with the principles of the Declaration of Helsinki.

## 3. Results

Of the 295 participants included in this study, 273 (93%) were classified into the skincare user group and 22 (7%) into the non-skincare user group. In terms of makeup use, 212 participants (72%) were classified as makeup users, whereas 83 (28%) were classified as non-makeup users. The usage rates of individual makeup products were as follows: foundation, 82%; lipstick, 57%; eyebrow products, 55%; eyeshadow, 28%; and blush, 28%.

Analyses of the participants’ makeup usage habits revealed significant differences in age (*p* = 0.001), grip strength (*p* = 0.011), one-leg standing time (*p* = 0.008), TUG score (*p* = 0.011), maximum walking speed (*p* = 0.022), GDS-5 score (*p* = 0.009), and EQ-5D-5L score (*p* = 0.049). Makeup users were significantly younger and demonstrated lower TUG scores, maximum walking speed, and GDS-5 scores, and significantly higher grip strength, one-leg standing time, and EQ-5D-5L scores compared with non-makeup users. After adjusting for age, ANCOVA revealed that makeup users had significantly lower GDS-5 scores (*p* = 0.008) and higher EQ-5D-5L scores (*p* = 0.038) compared with non-makeup users (Table 1).

When participants were further stratified according to the type of cosmetics used, significant differences were observed in age (*p* = 0.004) and TUG (*p* = 0.007), GDS-5 (*p* = 0.002), and EQ-5D-5L (*p* = 0.034) scores. The post hoc analysis using Tukey’s method revealed that individuals who used both lipstick and eyebrow products had significantly lower TUG and GDS-5 scores and significantly higher EQ-5D-5L scores compared with those who used neither (Table 2).

Multivariate logistic regression analysis was performed with age, maximum grip strength, MMSE, TUG, GDS-5, and EQ-5D-5L as independent variables. The items showing significant associations in both user groups were TUG (odds ratio 0.653, 95% confidence interval 0.448–0.952) and GDS-5 (odds ratio 0.592, 95% confidence interval 0.415–0.843) (Table 3). Furthermore, VIF values were calculated to confirm the absence of multicollinearity. VIF values ranged from 1.038 to 1.206, indicating no multicollinearity issues.

## 4. Discussion

This study investigated the characteristics of community-dwelling older women who habitually use makeup. The findings showed that the makeup users were younger and demonstrated superior grip strength, balance, maximum walking speed, depressive symptom scores, and HRQOL compared with non-makeup users. Notably, even after adjusting for age, the makeup group exhibited significantly better depression scores and HRQOL. These results suggest a positive association between makeup use and psychological functioning. Furthermore, participants who used both lipstick and eyebrow products showed significantly better dynamic balance, depressive symptom scores, and HRQOL. The results of the multinomial logistic regression analysis revealed a significant association with dynamic balance and depressive symptoms only in the group using both makeup items. Especially for older women, this finding suggests that the combined use of lipstick and eyebrow makeup is associated with favorable physical and psychological functioning.

In this study, 93% of the participants reported using skincare products. Previous studies have highlighted the role of skincare in improving skin moisture and maintaining overall skin health [28]. Previous studies have indicated that 90% of older adults use lotions [29], with a maximum usage rate of 72%. With regard to makeup use, the most commonly used products among participants were foundation (82%), lipstick (57%), eyebrow makeup (55%), eyeshadow (28%), and blush (28%). These findings are consistent with those of a previous study on older Japanese women, which reported usage rates of 83% for base makeup, 87% for lipstick, 56% for eyebrow products, and 28% for blush [29]. The lower rate of lipstick use observed in the present study may be attributed to reduced makeup application following the COVID-19 pandemic [30]. Nonetheless, the overall makeup habits of the participants were consistent with those reported in previous comparative studies, which showed that makeup users tend to be younger and exhibit lower TUG, maximum walking speed, and GDS-5 scores and higher grip strength, one-leg standing time (with eyes open), and EQ-5D-5L scores. A previous study indicated that makeup use decreases with age [10], a finding supported by the present study, which showed that participants in the makeup group were significantly younger than those in the non-makeup group. Handgrip strength, one-leg standing time (with eyes open), TUG score, and maximum walking speed have been associated with the frequency of going out [31,32]. Older women who habitually wear makeup may possess greater motivation to go out regularly [13,29]. The makeup group demonstrated superior grip strength, one-leg standing time (with eyes open), TUG scores, and maximum walking speed compared with the non-makeup group. However, the significant differences in these physical functions between the two groups were eliminated after adjusting for age. Previous studies have demonstrated that grip strength [33], one-leg standing time (with eyes open) [34], TUG score [35], and maximum walking speed [36] are influenced by age. Therefore, age was treated as a confounding variable in the present study, and the observed differences in physical function between the makeup and non-makeup groups were attributed to age.

The makeup group in the present study exhibited higher GDS-5 and EQ-5D-5L scores compared with the non-makeup group, even after adjusting for age. Veçoso et al. [12] reported that women who use makeup exhibit lower depression rates compared with non-users. Makeup use has been associated with increased social interaction, enhanced self-esteem [37], and greater happiness [38], which are protective factors against depression [39,40]. These findings may explain why older women who habitually wear makeup demonstrate lower GDS-5 scores and milder depression. Furthermore, Fujita and Wada [41] reported that makeup interventions improved HRQOL among older women residing in nursing homes, suggesting that makeup use can help maintain HRQOL in older women independent of their daily habits.

In the present study, comparisons of physical, cognitive, and psychological functions based on the types of makeup used revealed significant differences in age and TUG, GDS-5, and EQ-5D-5L scores. Post hoc analysis demonstrated that participants who used both lipstick and eyebrow products had significantly lower TUG and GDS-5 scores and significantly higher EQ-5D-5L scores compared with non-users, even after adjusting for age. Multivariate logistic regression analysis identified TUG and GDS-5 scores as factors associated with the types of cosmetics used, suggesting that these factors may influence not only psychological function but also physical function. Lipstick and eyebrow products are often regarded as enhancing perceived attractiveness and youthfulness [16,17]. Accordingly, their use may foster increased confidence and a stronger sense of self-affirmation in older women [37]. Previous studies have shown that higher levels of self-affirmation are associated with more frequent engagement in physical activity [42] and that physical activity contributes to improvements in dynamic balance [43]. The present study did not directly assess physical activity levels; however, older women who used both lipstick and eyebrow products likely maintained higher levels of physical activity, potentially contributing to improved dynamic balance and walking ability. Use of specific types of makeup, particularly lipstick and eyebrow products, may contribute to the improvement and maintenance of physical function in older women.

This study has some limitations. First, the study involved a relatively small sample size and focused on Japanese women, limiting the generalizability of the results. Future research should include larger, more diverse populations to enhance external validity. Additionally, the cross-sectional design of the study precludes any determination of causality between cosmetic use and physical, cognitive, and psychological functions. Longitudinal studies are warranted to clarify these potential relationships.

## 5. Limitations

As this was a cross-sectional observational study, a causal relationship between cosmetic use and physical, psychological, or cognitive function could not be established. Therefore, longitudinal studies or intervention studies are necessary to clarify causality. Furthermore, this study did not account for participant-specific factors, such as the presence of disease, household composition, or economic status, nor did it consider environmental factors. To more accurately investigate the relationship between cosmetics and health status, comprehensive studies incorporating these factors are necessary. Furthermore, this study was limited to elderly Japanese women; therefore, we could not clarify the relationship between cosmetic use and health status in women from different countries or cultural contexts. To identify differences across countries and cultures, the study population must be expanded.

## 6. Conclusions

Although no age-independent differences in physical function were observed between makeup users and non-users, older women who used makeup exhibited better psychological function. Furthermore, the use of both lipstick and eyebrow products was associated with superior psychological function and dynamic balance. These results indicate that makeup use may extend beyond aesthetic purposes and contribute to the maintenance of mental well-being and physical independence in older women.

## Figures and Tables

**Table 1:** Comparison of physical, cognitive, and psychological functions between habitual makeup users and non-makeup users (*n* = 295).

Variables	Make-Up Group (*n* = 212)	Non-Makeup Group (*n* = 83)	*t*-Test *p*-Value	ANCOVA *p*-Value
Age (years)	74.1 ± 5.3	76.3 ± 5.1	0.001	—
Grip strength (kg)	22.9 ± 3.6	21.7 ± 4.2	0.011	0.096
Sit-and-reach distance (cm)	35.7 ± 7.9	35.4 ± 10.1	0.778	0.854
One-leg standing time (s)	40.0 ± 35.6	28.3 ± 28.5	0.008	0.148
TUG (s)	5.9 ± 0.9	6.2 ± 1.1	0.011	0.208
Maximum walking speed (cm/sec)	189.1 ± 30.3	180.3 ± 27.5	0.022	0.154
MMSE (points)	28.3 ± 1.9	27.9 ± 2.1	0.12	0.285
GDS-5 (points)	0.6 ± 1.0	0.9 ± 1.1	0.009	0.008
EQ-5D-5L (points)	0.893 ± 0.099	0.866 ± 0.114	0.049	0.038

Values are expressed as the mean ± standard deviation. *p*-values were calculated using independent *t*-tests. ANCOVA was performed with age set as a covariate. Non-makeup group: Participants who use makeup less frequently. Abbreviations: TUG, timed up-and-go test; MMSE, Mini-Mental State Examination; GDS-5, Geriatric Depression Scale-5; EQ-5D-5L, EuroQol 5 Dimensions 5 Levels.

**Table 2:** Comparison of physical, cognitive, and psychological functions according to the type of makeup products used (*n* = 295).

Variables	Lipstick	Eyebrow Products	Both	Neither	ANOVA			ANCOVA		
	(a)	(b)	(c)	(d)						
	(*n* = 45)	(*n* = 40)	(*n* = 102)	(*n* = 108)	F-Value	*p*-Value	Post hoc	F-Value	*p*-Value	Post hoc
Age (years)	76.0 ± 5.6	72.7 ± 5.6	74.0 ± 4.9	75.6 ± 5.4	4.598	0.004	a > b, b < d	—	—	—
Grip strength (kg)	22.9 ± 3.4	23.3 ± 4.0	22.8 ± 3.8	22.0 ± 3.9	1.644	0.179	—	1.014	0.387	—
Sit-and-reach distance (cm)	35.0 ± 8.0	33.0 ± 8.7	37.0 ± 7.4	35.6 ± 9.4	2.305	0.077	—	2.339	0.074	—
One-leg standing time (s)	38.0 ± 36.5	36.6 ± 34.3	41.7 ± 35.4	31.5 ± 31.4	1.604	0.189	—	1.395	0.245	—
TUG (s)	6.0 ± 0.9	5.9 ± 1.0	5.7 ± 0.8	6.2 ± 1.1	4.166	0.007	c < d	3.085	0.028	c < d
Maximum walking speed (cm/sec)	187.4 ± 26.5	188.2 ± 35.6	191.3 ± 30.5	181.3 ± 27.4	2.053	0.107	—	1.366	0.253	—
MMSE (points)	28.3 ± 1.9	28.7 ± 1.9	28.3 ± 1.9	28.0 ± 2.0	1.276	0.283	—	0.808	0.490	—
GDS-5 (points)	0.8 ± 1.2	0.7 ± 1.0	0.4 ± 0.7	0.9 ± 1.1	5.094	0.002	c < d	5.153	0.002	c < d
EQ-5D-5L (points)	0.877 ± 0.119	0.891 ± 0.103	0.907 ± 0.089	0.866 ± 0.108	2.926	0.034	c > d	3.142	0.026	c > d

Values are expressed as the mean ± standard deviation. One-way ANOVA was used for group comparisons; post hoc tests were performed using Tukey’s method. ANCOVA was performed with age set as a covariate; post hoc comparisons were adjusted using the Bonferroni method. Lipstick (a): uses lipstick only. Eyebrow products (b): uses eyebrow products only. Both (c): uses both lipstick and eyebrow products. Neither (d): uses neither lipstick nor eyebrow products. Abbreviations: TUG, timed up-and-go test; MMSE, Mini-Mental State Examination; GDS-5, Geriatric Depression Scale-5; EQ-5D-5L, EuroQol 5 Dimensions 5 Levels.

**Table 3:** Multinomial logistic regression analysis of factors associated with cosmetic use categories.

						(*n* = 295)
	Lipstick		Eyebrows		Both	
	(*n* = 45)		(*n* = 40)		(*n* = 102)	
Variable	OR (95% CI)	*p*-Value	OR (95% CI)	*p*-Value	OR (95% CI)	*p*-Value
Age	1.04 (0.970–1.127)	0.248	0.91 (0.833–0.982)	0.017	0.97 (0.912–1.032)	0.334
Grip strength	1.07 (0.963–1.181)	0.219	1.05 (0.944–1.171)	0.358	1.00 (0.916–1.082)	0.920
TUG	0.85 (0.552–1.303)	0.452	1.14 (0.747–1.729)	0.551	0.65 (0.448–0.952)	0.027
MMSE	1.06 (0.889–1.272)	0.533	1.17 (0.947–1.444)	0.147	1.02 (0.883–1.185)	0.759
GDS-5	0.94 (0.662–1.328)	0.717	0.88 (0.600–1.269)	0.524	0.59 (0.415–0.843)	0.004
EQ-5D-5L	1.43 (0.039–51.836)	0.846	8.82 (0.158–491.944)	0.289	8.16 (0.375–177.470)	0.181

Odds ratios (OR) with 95% confidence intervals (CIs) are reported. Model chi-square < 0.001. The reference category for the dependent variable is “Neither.” Lipstick: used lipstick only. Eyebrows: used eyebrow products only. Both: used both lipstick and eyebrow products. Neither: used neither lipstick nor eyebrow products. Abbreviations: TUG, Timed Up & Go test; MMSE, Mini-Mental State Examination; GDS-5, Geriatric Depression Scale 5-item version; EQ-5D-5L, EuroQol 5 Dimensions 5 Levels.

## Data Availability

The data presented in this study are available on request from the corresponding author. The data are not publicly available due to privacy.

## Abbreviations

The following abbreviations are used in this manuscript:

TUG	timed up-and-go test
MMSE	Mini-Mental State Examination
GDS-5	Geriatric Depression Scale-5
EQ-5D-5L	EuroQol 5 Dimensions 5 Levels
